# Communication Training For Medical Professionals: Proof Of Concept

**DOI:** 10.1002/alz.094778

**Published:** 2025-01-09

**Authors:** Nancy L Brown

**Affiliations:** ^1^ Validation Training Institute, Jerusalem, Israel; University of Edinburgh, Edinburgh United Kingdom

## Abstract

**Background:**

Despite the prevalence of patients living with dementia, physicians and medical students, in particular, have received little or no dementia‐specific training to help them communicate effectively with this population. Connecting and communicating with persons living through cognitive change requires specific skills: developing empathy and identifying coping mechanisms, symbols, and the stages of resolutions for those living with cognitive decline. These skills were taught in the beta test, Validation for Physicians and Medical Provider training, based upon the evidence‐based Validation method by Naomi Feil.

**Method:**

The pilot program included physicians and other healthcare professionals. In this single‐group design, pre‐ and post‐surveys consisted of a knowledge scale and SRDB (self‐efficacy) ranked on a Likert scale of 1 to 5 and analyzed using SPSS. Post‐training individual interviews concluded the beta‐testing. Thirty professionals enrolled in the training; eight participants completed every aspect of the beta‐testing.

**Result:**

Despite the small sample size, the validated measurements used showed trends approaching significance in knowledge and self‐efficacy. Participants highly valued the training’s content and pedagogy.

**Conclusion:**

Technical problems were the main reason for attrition. Overall, the Validation method was found useful in practice and improved confidence, knowledge, and communication behaviors. Improvements are needed in the choices for validated measures and the online platform. “Validation has made me a better doctor. It has changed my perspective on aging” (participant).

